# Reconstructing the age and historical biogeography of the ancient flowering-plant family Hydatellaceae (Nymphaeales)

**DOI:** 10.1186/1471-2148-14-102

**Published:** 2014-05-13

**Authors:** William J D Iles, Christopher Lee, Dmitry D Sokoloff, Margarita V Remizowa, Shrirang R Yadav, Matthew D Barrett, Russell L Barrett, Terry D Macfarlane, Paula J Rudall, Sean W Graham

**Affiliations:** 1 Department of Botany, University of British Columbia, 3529-6270 University Blvd, Vancouver, British Columbia V6T 1Z4, Canada; 2 UBC Botanical Garden & Centre for Plant Research, University of British Columbia, 6804 Marine Dr SW, Vancouver, British Columbia V6T 1Z4, Canada; 3 Department of Higher Plants, Biological Faculty, M.V. Lomonosov Moscow State University, 119234 Moscow, Russia; 4 Department of Botany, Shivaji University, Kolhapur 416 004 Maharashtra, India; 5 Botanic Gardens and Parks Authority, Kings Park and Botanic Garden, West Perth, WA 6005, Australia and School of Plant Biology, University of Western Australia, Nedlands, WA 6009, Australia; 6 Department of Parks & Wildlife, Western Australian Herbarium, Science Division, Brain Street, Manjimup 6258 WA, Australia; 7 Jodrell Laboratory, Royal Botanic Gardens, Kew, TW9 3AB, Richmond, Surrey, UK

**Keywords:** Aquatic plants, Austral, Ephemeral habitats, Extinction rates, Intercontinental dispersal, ANITA-grade angiosperms, *Trithuria*, Vicariance

## Abstract

**Background:**

The aquatic flowering-plant family Hydatellaceae has a classic Gondwanan distribution, as it is found in Australia, India and New Zealand. To shed light on the biogeographic history of this apparently ancient branch of angiosperm phylogeny, we dated the family in the context of other seed-plant divergences, and evaluated its biogeography using parsimony and likelihood methods. We also explicitly tested the effect of different extinction rates on biogeographic inferences.

**Results:**

We infer that the stem lineage of Hydatellaceae originated in the Lower Cretaceous; in contrast, its crown originated much more recently, in the early Miocene, with the bulk of its diversification after the onset of the Pliocene. Biogeographic reconstructions predict a mix of dispersal and vicariance events, but considerations of geological history preclude most vicariance events, besides a split at the root of the family between southern and northern clades. High extinction rates are plausible in the family, and when these are taken into account there is greater uncertainty in biogeographic inferences.

**Conclusions:**

A stem origin for Hydatellaceae in the Lower Cretaceous is consistent with the initial appearance of fossils attributed to its sister clade, the water lilies. In contrast, the crown clade is young, indicating that vicariant explanations for species outside Australia are improbable. Although long-distance dispersal is likely the primary driver of biogeographic distribution in Hydatellaceae, we infer that the recent drying out of central Australia divided the family into tropical vs. subtropical/temperate clades around the beginning of the Miocene.

## Background

Australia has seen widespread rainforest replaced with deserts, savannah and sclerophyll biomes since the Eocene, in response to global cooling [1]. Despite the dramatic loss of mesophytic habitat, it has a well-developed wetland flora, with many endemic species [2]. Perhaps the most unique of these habitats are ephemeral bodies of water that are home to communities characterized by extreme reduction in plant size, and annual or geophytic life histories [3,4]. Common Australian members of this ephemeral aquatic habitat include Centrolepidaceae (a family closely related to or possibly embedded within the southern rushes [5], Restionaceae), the sundew genus *Drosera* L. (Droseraceae), and *Hydrocotyle* L. (Araliaceae) [3,6], but its most noteworthy component may be the family Hydatellaceae [7]. Most members of Hydatellaceae exemplify the ephemeral aquatic syndrome, apart from a recently derived pair of perennial apomictic species that live submerged in more permanent bodies of water [8,9].

Hydatellaceae were recently recognized as the sister group of the water lilies (Cabombaceae and Nymphaeaceae), placing their divergence close to the root of angiosperm phylogeny e.g., [10]. They have since attracted considerable attention because of the insights they may provide into the evolution of early angiosperms [11-16]. Of particular interest is the nature of the reproductive structures in the family, which may represent floral, prefloral, or pseudanthial arrangements of reproductive organs, and the incidence of unisexual and bisexual reproductive units. These may bear on our understanding of the ancestral floral *Bauplan* of angiosperms [8,9,13]. Contemporary taxonomic and phylogenetic work on the family recognizes one genus, *Trithuria* Hook. f., and 12 species in four monophyletic sections [8,9].

**Figure 1:**
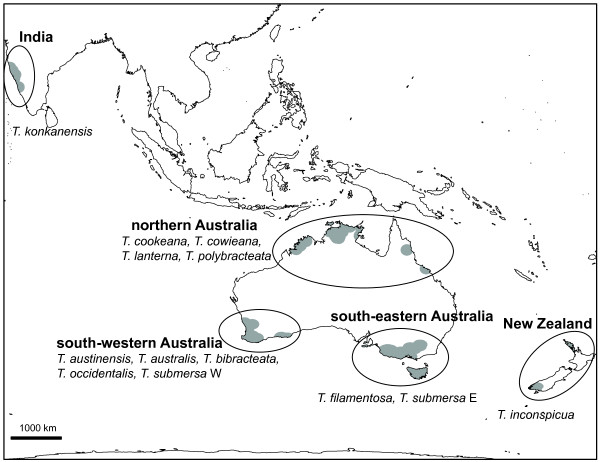
**Global distribution of Hydatellaceae.** The distribution is based on online herbarium resources [19] and new collection records; each collection locality is represented by a 120 km radial sweep. Five biogeographic areas are delineated by labelled ovals: India, New Zealand, northern Australia, south-eastern Australia, and south-western Australia. Species found in each area are listed; note that *Trithuria submersa* is treated here as south-eastern and south-western species. We created the map using ArcMAP version 9.3 (Environmental Systems Research Institute, Redlands, CA, USA); the projection is Lambert’s cylindrical equal area.

The distribution of the Southern Hemisphere biota has been traditionally framed in terms of vicariance events resulting from the breakup of Gondwana [17]. However, recent studies have suggested a more important role for long-distance dispersal, especially in plants [17]. Within Australia, the constriction and fragmentation of mesophytic and rainforest habitats since the Eocene [1] have led to congruent patterns of vicariant speciation across a number of plant lineages [18]. Hydatellaceae display a classic Gondwanan distribution, being present in Australia, India and New Zealand (Figure 1; the distribution is based on online herbarium records [19] and new collection records), which may imply a relictual intercontinental distribution and great antiquity for the crown clade [20]. While vicariant processes at both the hemispheric and continental scale could explain the extant distribution of Hydatellaceae, this distribution may also result from long-distance dispersal [9,17] (the wide and potentially trans-oceanic distribution of many aquatic plant species is widely suspected to be facilitated by the enhanced dispersal capabilities of their diaspores, often considered to be due to water-fowl vectors [21]). Evaluating these biogeographic hypotheses requires placing geological and geographical events in the context of dated phylogenies [22]. Most organisms lack an extensive fossil record, and so molecular dating analyses use age information from parts of the phylogeny with a good fossil record to inform the age of nodes that lack it [23].

Since the recognition that Hydatellaceae represents an ancient angiosperm lineage, a few fossils have been linked with it [7,24-26]. The most spectacular of these may be the aquatic plant *Archaefructus*, represented by whole fruiting plants from the Yixian Formation of Liaoning, China [27,28]. However, the timing and interpretation of these and other records remains contentious [7,29]. Unlike the fossil record of Hydatellaceae, water lilies have an extensive record that extends to the Lower Cretaceous [29-31]. Collectively these fossils suggest that aquatic niches were exploited early in the evolution of angiosperms, although the aquatic life-form is unlikely to be ancestral in flowering plants as a whole [32,33]. Nonetheless, an improved understanding of the diversification of Hydatellaceae may help illuminate early angiosperm ecology and how plants colonize ephemeral wetlands, which represent a unique and potentially stressful environment [4,34].

To address these questions we dated the earliest splits in Hydatellaceae using 17 plastid-genes sampled from across the seed plants, and used the resulting posterior age distributions as secondary calibrations for a species-tree analysis of the entire family, which lacks suitable fossil calibrations. Although the use of secondary calibration points has been criticized for propagating “error free” values into downstream analyses [35], here we use the entire posterior distribution from the seed-plant analysis as a prior for the subsequent analysis, accounting for the associated uncertainty [36]. We used the dated species tree to explore biogeographic hypotheses using parsimony and likelihood. In particular, likelihood-based approaches allow the estimation of parameters such as speciation rate. We also explicitly test the effect of extinction rate on biogeographic reconstruction, as this may be high in Hydatellaceae due to the patchy distribution of their habitat in space and time [4,34] and is also suggested by the “broom-and-handle” shape of the phylogeny [9,37-39].

## Methods

### Fossil selection and molecular dating

Fossils with unequivocal affinity to Hydatellaceae are unknown [7]. We therefore first estimated the crown-age of the family in a seed-plant analysis with 17 exemplar taxa constrained by eight fossils (Table 1; see [24,40-52]). We added *Trithuria cowieana* D.D. Sokoloff, Remizowa, T.D. Macfarl. & Rudall (*Macfarlane & al. 4217*, MW; [GenBank: JQ284074, JQ284187, JQ284224, KJ725347–KJ725349]) to an existing data set that included *T. filamentosa* Rodway and *T. submersa* Hook. f. [10,53,54] as these three species define the deepest phylogenetic splits in Hydatellaceae [9]. This seed-plant matrix includes three gymnosperms and major lineages of angiosperms [53]. Genomic sampling focused on 13 single-copy plastid genes (comprising four multi-gene clusters, *psb*D–*psb*C, *psb*E–*psb*F–*psb*L–*psb*J, *psb*B–*psb*T–*psb*N–*psb*H, and three single-gene regions, *ndh*F, *rbc*L, and *atp*B). Only protein-coding regions were considered. Details of DNA extraction, amplification, sequencing, contig assembly and alignment are described elsewhere [9,10,55,56]; for a list of accessions and the alignment matrix, see Additional files 1 and 2, respectively.

**Table 1:** **Fossil calibrations for seed**-**plant phylogeny**

**Node**	**Calibration**	**Fossil**	**Age(Ma)**	**Priors**	**References**
A	Crown seed plants	Cordaites	315	316.0–367.5 (2, 1)	[40,41]
C	Stem angiosperms	Glossopterid, *Gangamopteris* McCoy	293.8	294.8–346.3 (2, 1)	[24,40,42,43]
G	Stem Cabombaceae	*Pluricarpellatia peltata* B. Mohr, Bernardes-de-Oliveira & D.W. Taylor	98.7*	99.1–118.0 (1, 1)	[44,45]
H	Crown Nymphaeaceae	*Monetianthus mirus* Friis, Pedersen, von Balthazar, Grimm & Crane	92.8*	93.2–112.1 (1, 1)	[46,47]
M	Stem Trimeniaceae	Unnamed seed	98.7	98.9–110.4 (0.5, 1)	[48]
N	Stem eudicots	Tricolpate pollen	124*	124.2–135.7 (0.5, 1)	[49,50]
O	Stem Araceae	*Mayoa portugallica* Friis, Pedersen & Crane	96.1*	96.5–115.4 (1, 1)	[51]
P	Stem Platanaceae	West Brothers platanoid and *Sapindopsis* Fontaine	92.8*	93.2–112.1 (1, 1)	[52]

‘Node’ refers to placement on phylogeny in Additional file 3. Fossil ages follow the references, except that some ages are modified following [49], indicated with an asterisk. Calibration priors are lognormal; the 95% prior interval is given, followed by the lognormal mean and standard deviation in parentheses.

To test for and accommodate non-clocklike behaviour in the seed-plant data set we used the Bayesian random local clocks (RLC) method [57]. This accommodates molecular rate variation by allowing different sub-branches of the tree to have unique molecular clocks. Dornberg et al. [58] examined the performance of this method against the more widely used uncorrelated lognormal (UCLN) method [59] for real and simulated data sets that show high amounts of inter-clade rate variability, and found that the RLC model performed better in the presence of clade-specific rate shifts. This may be pertinent to angiosperm studies like ours, as there are known to be substantial shifts in rates among major angiosperm clades that are associated with changes in habit and life history [60]. In particular, Hydatellaceae occupy a part of the tree where there were multiple shifts in habit (for example, Hydatellaceae are mostly herbaceous annuals, water lilies are mostly perennial herbs, *Amborella* Baill. and Austrobaileyales include shrubs, small trees and lianas). The method is implemented in BEAST version 1.6.1. We used a GTR + Γ model of sequence evolution, with default priors (or those suggested by http://code.google.com/p/beast-mcmc/wiki/ParameterPriors if not automatically implemented). The BEAST analysis requires that each of the fossil calibrations have an associated prior. We used lognormal priors with 95% prior intervals of ~10–20% of the fossil age (Table 1), consistent with some other studies, e.g., [61] (the RLC method is also more robust than the UCLN method to variation in the width of the 95% prior interval [58]). We ran seven runs of 4.0 × 10^7^ generations, and considered four that converged on the same posterior and likelihood scores after 10% burnin. The estimated sample sizes of run statistics (posterior, prior, likelihood, parameter estimates) were all over 200 when these runs were pooled. The seed-plant chronogram and a table of divergence times is presented in Additional file 3, and the tree file is provided in Additional file 4. In all analyses we constrained Nymphaeaceae s.s. (i.e., excluding Cabombaceae) to be monophyletic, consistent with molecular and morphological analyses [62-64]. We also tested a constraint that forces *Amborella* to be the sister group of all other angiosperms; this arrangement contrasts with a clade comprising *Amborella* and Nymphaeales that we recovered in the RLC analysis, see below (these two alternative arrangements have been recovered in different studies, see [53], for example). We constrained cycads to be the sister group of angiosperms among extant seed plants, consistent with some recent studies [24,42,43,54], but also explored alternative gymnosperm sister groups to angiosperms (conifers alone, *Ginkgo* L. alone, or pairwise combinations of conifers, cycads and *Ginkgo*), or used no outgroup constraints. For these different constraint analyses we ran a single 4.0 × 10^7^ generation replicate; they all indicated only a minimal effect on the two ages within Hydatellaceae (<1 Myr difference; data not shown).

To date the Hydatellaceae species tree we considered the data set of [9], which consists of two unlinked loci (four plastid regions and the nuclear ribosomal internal transcribed spacer region, ITS) for all species except *Trithuria occidentalis* Benth. which was only sampled for one plastid region. In all analyses *T. submersa* was provisionally considered to comprise separate eastern and western species, following [9]. The data were analysed with *BEAST, which estimates the species tree with a Bayesian implementation of the multi-species coalescent [65]. We used the settings outlined in [9], with the exception that we assigned the two Hydatellaceae posterior distributions determined from the RLC seed-plant analysis (see Additional file 3) as Gaussian priors for the corresponding splits in the species tree (i.e., the crown node of Hydatellaceae and the crown node of the clade consisting of sect. *Hydatella* and sect. *Trithuria*). These priors were only applied to the plastid loci (for which there was outgroup data), using the rooting of Hydatellaceae determined in the seed-plant analysis (see Additional file 3).

### Biogeographic reconstructions

**Figure 2:**
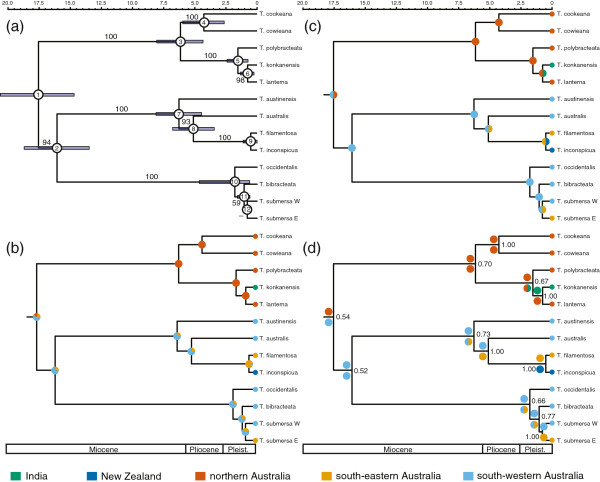
**Dated species tree and biogeographic inferences for Hydatellaceae. (a)** Timing of divergences in Hydatellaceae inferred using a multi-species coalescent analysis, based on four plastid genes and nuclear ITS regions, with prior dating estimates for the first two nodes derived from a separate seed-plant analysis (see Additional file 3; labelled nodes are referred to in Additional file 5). Numbers beside branches are support values (posterior probabilities expressed as percentages); dashes indicate <50% support. Divergence time uncertainty is noted by blue bars, representing 95% HPD. The time scale is in Ma. Letters adjacent to tips represent: E = east, T. =*Trithuria*, W = west. Historical biogeography inferred in **(b)** with the full model of ML ancestral-state reconstruction, in **(c)** with MP based dispersal-vicariance analysis, and in **(d)** with ML based dispersal-extinction cladogenesis analysis. Pie fractions in **(b)** represent relative likelihoods; in **(c)** and **(d)** they represent joint areas where the species is inferred to have existed in multiple areas. The relative likelihood of the best geographic range pair is shown in **(d)** adjacent to individual nodes.

We reconstructed ancestral areas using three methods: maximum parsimony dispersal-vicariance (DIVA; [66]), maximum likelihood (ML) dispersal-extinction-cladogenesis (DEC; [67]), and ML ancestral-state reconstruction (ASR; cf. [68]). The DIVA and DEC methods allow the range of extant species and internal nodes (ancestral species) to encompass multiple discrete areas, and identify dispersal, vicariance or area extinction events. When vicariance events predicted in these analyses could not be explained by contemporaneous geographic division, we treated them as indicative of long-distance dispersal events. In contrast, ASR implicitly only considers dispersal/extinction, and restricts each species range and internal node to a single area. In all cases we used the species tree as the reference phylogeny (Figure 2a) and considered the five major biogeographic areas that define the range of Hydatellaceae: (1) India; (2) northern Australia; (3) New Zealand; (4) south-eastern Australia; (5) south-western Australia (Figure 1). We treated south-eastern Australia as one area, despite it being climatically variable, because species ranges there (*Trithuria filamentosa* and *T. submersa*) are essentially contiguous within Tasmania, and there is no evidence of a strong geographic barrier between them [8]. We used RASP version 2.0 Beta [66,69] to perform the DIVA analysis, and for DEC we used Lagrange version 20110117 (http://www.reelab.net/lagrange/configurator/index; [67]). We constrained some of the area connectivities for the DEC analysis, following the advice of the Lagrange website, which we based on our understanding of niche profiles of the species (unpublished data) and the underlying phylogenetic relationships. In particular, we constrained the connectivity of India to northern Australia only, and of New Zealand to south-eastern Australia only. To make comparisons more meaningful between methods we also constrained the DIVA analysis in the same way. In addition, for the DEC analysis we considered all dispersal paths to have the same rate (as the allowed distances are comparable in magnitude; ~1500–6500 km), so we set all values in the dispersal rate scaling matrix to 1.0 (i.e., all dispersal path rates are multiplied by “1”). Simulation studies show that area extinction rates in Lagrange are strongly biased towards zero [67]; as an alternative we evaluated lineage (species) extinction rates [70] as a proxy for the area extinction rate, the parameter used in our analyses (species extinction can be thought to occur when all the areas that encompass the species range go extinct, and may be an underestimate of the area extinction rate). We used the R package Diversitree version 0.7-2 [71,72] to evaluate extinction and speciation rates on the Hydatellaceae species tree alone, or on the species tree with the addition of one outgroup separated by 127 Myr (see below). Based on the results of the extinction-speciation analysis we chose six area extinction rates (0.001, 0.01, 0.05, 0.1, 0.5, and 1.0 Myr^-1^) spanning the range of lineage extinction rates to explore the effect of different rates on biogeographic reconstructions. Dispersal rates were iteratively optimized in Lagrange for each extinction rate.

The ASR analyses were performed with BayesTraits version 1.0 (http://www.evolution.rdg.ac.uk; [73]). We considered three nested models which were evaluated using the corrected Akaike information criterion (AICc; [74]). The most complex model (hereafter the ‘full model’) assumed three separate symmetrical transition rates: between Australia and India or New Zealand (assuming trans-oceanic dispersals to be equivalent), between south-western and south-eastern Australia (dispersals across the Nullarbor Plain), and between northern Australia and south-western or south-eastern Australia (dispersals across the arid zone). The simplest model (‘simple model’) consists of a single rate between all the allowed transition rates in the full model. The two-rate transition model has symmetric rates between Australia and India or New Zealand, contrasting with a separate rate for all transitions within Australia (‘continental model’). Root state frequencies were set to empirical values.

## Results

### Molecular dating and diversification

Hydatellaceae are estimated to have diverged from the water lilies 126.7 Ma (120.6–133.2 Ma, 95% HPD), in the Lower Cretaceous, with a crown clade age of 19.1 Ma (15.7–23.4 Ma, 95% HPD), in the early Miocene (see Additional file 3). The estimated multi-species coalescent age for the crown of Hydatellaceae is 17.6 Ma (14.7–20.6 Ma, 95% HPD), in the early Miocene, with most diversification occurring after ~6 Ma, in the late Miocene (Figure 2a; see Additional file 5 for a table of divergence times, and Additional file 6 for the tree file). For the speciation-extinction analysis we estimated a speciation rate of 0.430 Myr^-1^ (0.107–0.881 Myr^-1^; 95% HPD) and a lineage extinction rate of 0.446 Myr^-1^ (0.003–0.955 Myr^-1^; 95% HPD). Including a distantly related outgroup did not substantially change speciation or extinction parameter estimates (data not shown).

### Biogeographic reconstructions

The full ASR model had the best AICc score (Figure 2b; differences between best and alternative models: simple Δ = 1.06; continental Δ = 2.135). It shows a split between the tropical (northern Australia and India) and subtropical/temperate (south western Australia, south eastern Australia, and New Zealand) clades (Figure 2b). Within the tropical clade we infer that the Indian species, *Trithuria konkanensis* S.R. Yadav & Janarth, represents a relatively recent long-distance dispersal event from northern Australia (Figure 2b). Within the subtropical/temperate clade, the New Zealand species *T. inconspicua* represents a long-distance dispersal event from south-eastern Australia. There is no significant support for a particular direction of dispersal between south-western and south-eastern Australia (Figure 2b).

**Figure 3:**
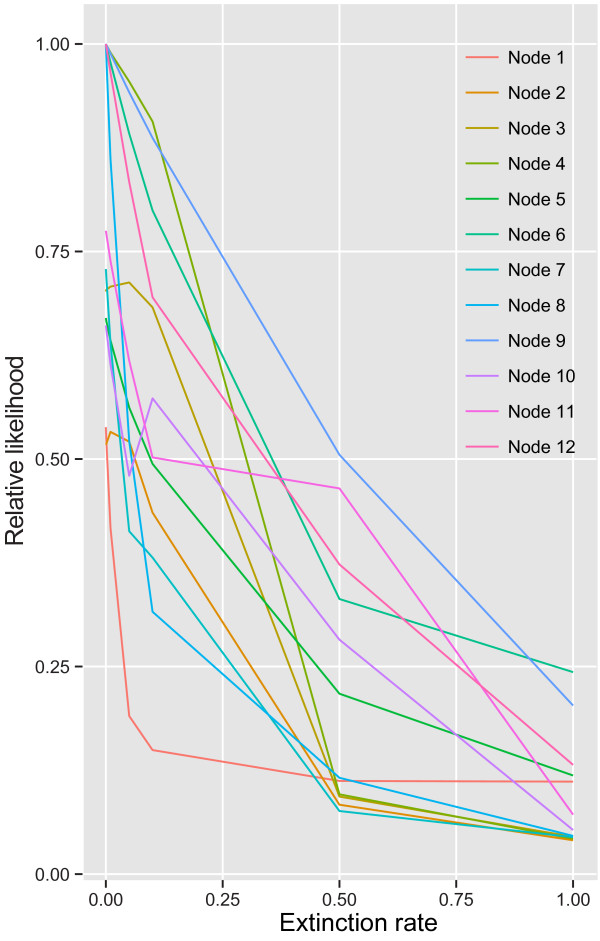
**Relationship between area extinction rate and relative likelihood of range pairs in the dispersal-extinction-cladogenesis(DEC)analysis.** The plot depicts the value of the range pair with the largest relative likelihood at each node and extinction rate; note that the top pair of ranges is not necessarily consistent across extinction rates. The ‘zero’ extinction value is the auto-optimized estimate (4.285 × 10^-9^ Myr^-1^). Node numbers correspond to those in Figure 2a.

**Figure 4:**
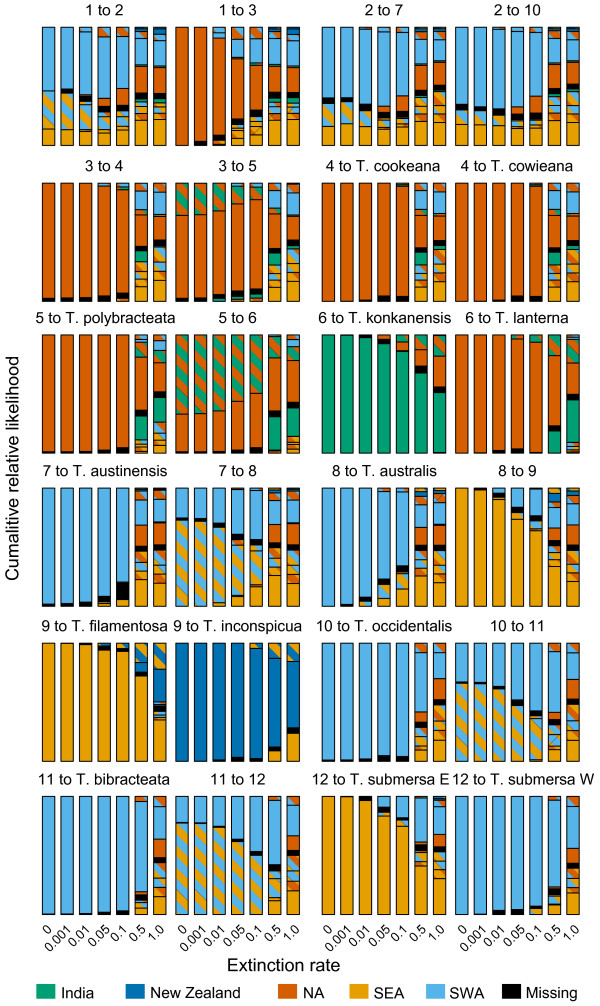
**Relationship between extinction rate and relative likelihood of geographic ranges in each descendant lineage of a range-pair in the dispersal-extinction-cladogenesis(DEC)analysis.** The likelihoods sum to unity in each bar; subdivisions represent the relative likelihood of each range. Hashed ranges comprise more than one area. NA, northern Australia; SEA, south-eastern Australia; SWA, south-western Australia. The ‘zero’ extinction rate is the auto-optimized estimate (4.285 × 10^-9^ Myr^-1^). Node numbers correspond to those in Figure 2a; the range pair depicted in Figure 2d reports the best pair descending from each node (e.g., node 1 to 2 and 1 to 3) at the auto-optimized rate.

The DIVA analysis reconstructed several vicariance events across the phylogeny (Figure 2c). There is an inferred vicariance event at the root of the family, between the tropical and subtropical/temperate clades (Figure 2c). An additional one was predicted between northern Australia and India in the tropical clade, and in the subtropical/temperate clade two more were predicted between south-western Australia and south-eastern Australia, and one between south-eastern Australia and New Zealand (Figure 2c). The DEC analysis recovered similar patterns of vicariance across Hydatellaceae, although it also inferred dispersals, for example from south-eastern Australia to New Zealand (Figure 2d). In both analyses, vicariance is implausible in the context of dating analyses, except for the one involving the root split between the tropical and subtropical/temperate clade. Increasing the extinction rate in the DEC analysis served to depress confidence in the estimated ancestral ranges for all nodes, considering both the relative likelihood of the best reconstructed range pair (Figure 3) and the relative likelihood of the individual ranges at nodes (Figure 4).

## Discussion

The phylogenetic origin of Hydatellaceae near the root of angiosperm phylogeny [10] and lack of reliable fossils [7] make consideration of the family age infeasible outside the context of angiosperm divergence times. Unfortunately the crown age and subsequent timing of diversification of angiosperms remains one of the most vexing questions in evolutionary biology, with some molecular estimates [42,49,61] substantially older (~100 Myr) than the oldest reported crown angiosperm fossils [29]. Our estimated age of 158.7 Ma (151.0–167.7 Ma, 95% HPD; see Additional file 3) is more in-line with less extreme results reported elsewhere [75,76]. A stem age for Hydatellaceae of ~127 Ma (see Results and Additional file 3) suggests that stem lineage Hydatellaceae were colonizing aquatic environments in the Lower Cretaceous, although when Hydatellaceae acquired the unique suite of traits suited for ephemeral aquatic habitats is unclear.

A crown age for Hydatellaceae in the early Miocene (~18 Ma, see Figure 2a and Additional file 5) indicates that a proposed Gondwanan explanation for the current intercontinental distribution [20] is incorrect, as it would require that the Indian and north Australian species pair *Trithuria konkanensis* and *T. lanterna* diverged ~125 Ma, according to the timing of the breakup of East Gondwana [77], instead of the estimated divergence time of 0.76 Ma (0.24–1.33 Ma, 95% HPD; see Figure 2a and Additional file 5). This highlights the importance of assessing proposed vicariant patterns with a careful consideration of phylogeny, geology, and estimated divergence times [22].

Within Australia, climate driven vicariance events are more plausible, although here as well, the last submersion of the Nullarbor Plain (~15 Ma), which separates the south-eastern and south-western regions, substantially predated the relevant phylogenetic splits (Figure 2a; [18]). However, the DIVA and DEC analyses indicate a continent-scale vicariance event at the root of extant Hydatellaceae (Figure 2c,d). The interior of Australia was still relatively wet in the early Miocene (up to the mid-Miocene), and although there were permanent lakes, there was also a marked dry season, indicating the potential for ephemeral aquatic habitats [1]. The continued aridification of central Australia presumably led to this vicariance event. Our analyses therefore support a minimum of four long-distance dispersal events in Hydatellaceae (Australia to India, Australia to New Zealand, and two instances from south-western to south-eastern Australia; Figure 2). The inferred long-distance dispersal events likely involved selfers or apomicts, consistent with Baker's Law [78]. The New Zealand species *Trithuria inconspicua* and its Tasmanian sister species *T. filamentosa* are both thought to be perennial apomicts [79,80]; selfing is thought to characterize the Indian *T. konkanensis* and its sister species, *T. lanterna*, in northern Australia [8,81]. Baker’s Law has been extended to dispersal in general, not just islands, and as a result we expect selfing taxa to have wider distributions than outcrossing ones [82]. This seems to be the case in Hydatellaceae, where dioecious species are generally much more limited in distribution than related cosexual species [8].

Statistical biogeographic methods such as DEC allow not only an examination of the biogeographic history of a clade and an estimate of the processes involved in producing that history (dispersal, vicariance and extinction), but also quantification of how confident we are in these reconstructions, via consideration of (relative) likelihoods. A strong bias towards estimating zero area extinction rates may occur in the DEC framework, both for real and simulated data sets [67]. We examined the effect that this may have on our reconstructions by manually varying the extinction rate based on the range of values seen in our speciation-extinction analysis (see Results). Our confidence in reconstructing both (a) range pairs (thereby indicating possible processes such as vicariance or dispersal; Figure 3), and (b) each individual descendent lineage’s range (as indicated by the relative likelihoods for each range across all possible range pairs; Figure 4), is compromised at higher rates. For the estimated extinction rate based on tree shape (~0.5 Myr^-1^), there is very little confidence in any particular range pair (relative likelihoods are <0.6, Figure 3), and in the ranges of individual descendent lineages, besides a few of the very shallowest and youngest nodes (Figure 4). Estimating extinction rates from phylogenies is contentious and often leads to large confidence intervals [37,83,84], which is what we infer with our data. Nevertheless, our results are potentially in line with estimates for other herbaceous groups [85]. Even relatively moderate extinction rates may limit our ability to confidently reconstruct biogeographic history, and so inferences based on the very low optimal extinction rate predicted in the DEC analysis should be treated cautiously. However, despite the greater uncertainty in biogeographic reconstructions at higher extinction rates, the New Zealand and Indian species must represent recent long-distance dispersal events, given their very recent separation from closely related Australian species. The Indian species was discovered only recently (1994; [20]) and yet has a relatively extensive range [20], which may add further weight to the possibility that the global distribution of the family may be more extensive than is currently reported [7,10,86]. Further phylogeographic work in individual species may also reveal additional instances of intra-specific migration and extinction (e.g., with regards to the substantially disjunct distribution of *Trithuria inconspicua* in New Zealand).

## Conclusions

Our analyses suggest the Hydatellaceae lineage arose in the Lower Cretaceous, but that extant species diversity dates from the Miocene. The former age highlights the early exploitation of aquatic environments by angiosperms. Our results also emphasize the potentially high extinction rate associated with ephemeral aquatic habitats. Despite having a classical Gondwanan intercontinental pattern, the young age of the crown clade of Hydatellaceae contradicts the role of vicariance events in shaping the family’s distribution. This suggests instead that long-distance dispersal is predominately responsible for its disjunct distribution both within and outside Australia.

## Availability of supporting data

The new data sets supporting the results of this article are included within the article (and its additional files).

## Abbreviations

AICc: Corrected Akaike information criterion; ASR: Ancestral state reconstruction; BI: Bayesian inference; DEC: Dispersal extinction cladogenesis; DIVA: Dispersal vicariance; HPD: Highest posterior density; Ma: Millions of years ago; ML: Maximum likelihood; Myr: Millions of years; RLC: Random local clock; UCLN: Uncorrelated lognormal.

## Competing interests

The authors declare that they have no competing interests.

## Authors' contributions

WJDI designed the study and carried out the analyses. CL constructed the distribution map. All authors contributed to drafting the manuscript; writing was led by WJDI and SWG. All authors read and approved the final manuscript.

## Supplementary Material

Additional file 1 Voucher and accession information for seed-plant molecular dating. Click here for file

Additional file 2 Seed plant alignment matrix. Click here for file

Additional file 3 Chronogram and table of inferred ages from the Bayesian random local clock molecular dating analysis of the seed plants. Click here for file

Additional file 4 Tree file for the seed-plant Bayesian random local clock tree. Click here for file

Additional file 5 Table of inferred ages in the Bayesian multi-species coalescent molecular dating of Hydatellaceae. Click here for file

Additional file 6 Tree file for the Hydatellaceae Bayesian multi-species coalescent tree. Click here for file
