# Supplementary material for: Reconstructing the age and historical biogeography of the ancient flowering-plant family Hydatellaceae (Nymphaeales)
Source: BMC Evol Biol. 2014 May 13;14:102. doi: 10.1186/1471-2148-14-102 (PMC4030046; doi:10.1186/1471-2148-14-102)
Supplement: Additional file 1 — Voucher and accession information for seed-plant molecular dating. [file 1471-2148-14-102-S1.doc]

**Additional file 1.** List of species and GenBank accessions used in seed-plant dating. Taxon, collection and herbarium (Thiers, continuously updated) if newly sequenced, GenBank accession numbers for *atp*B, *ndh*F, *psb*BTNH, *psb*DC, *psb*EFLJ, and *rbc*L unless otherwise noted. Only *Trithuria cowieana* is in part newly sequenced (indicated by an asterisk).

***Acorus calamus*** L., AJ235381, AY007647, AF123843, AF123813, AF123828, D28865. ***Amborella trichopoda*** Baill., AF235041, AF235046, AF235042, AF235043, AF235044, L12628. ***Austrobaileya scandens*** C.T.White, AF092107, AF238052, AY007460, AF239777, AY007475, L12632. ***Cabomba caroliniana*** A.Gray, AF187058, AF123801, AF123845, AF123815, AF123830, M77027. ***Cycas revoluta*** Thunb., AF469657, AF469695, AF469707, AF462403, AF469716, AF462411. ***Ginkgo biloba*** L., AJ235481, AF123807, AF123851, AF123821, AF123836, D10733. ***Nandina domestica*** Thunb., complete plastid genome NC_008336. ***Nuphar advena*** Ait., complete plastid genome NC_008788. ***Nymphaea odorata*** Ait., AJ235544, AF188853, AF188851, AF188854, AF188852, M77034. ***Pinus thunbergii*** Parl., complete plastid genome D17510. ***Platanus occidentalis*** L., complete plastid genome NC_008335. ***Schisandra chinensis*** (Turcz.) Baill., AF239790, AF238062, AY007470, AF239791, AY007485, AF238061. ***Spathiphyllum wallisii*** Regel, AJ235606, AY007658, AY007471, AF239794, AY007487, AJ235807. ***Trimenia moorei*** (Oliv.) Philipson, AY116653, AY116655, AY116656, AY116657, AY116652, AY116658. ***Trithuria cowieana*** D.D.Sokoloff, Remizowa, T.D.Macfarl. & Rudall, *Macfarlane & al. 4217* (MW), JQ284187, JQ284074, KJ725347*, KJ725348*, KJ725349*, JQ284224. ***Trithuria filamentosa*** Rodway, FJ514801, FJ514806, FJ514802, FJ514803, FJ514804, FJ514807. ***Trithuria submersa*** Hook.f., AJ419142, AF547020, DQ915189, EF153940, EF153946, DQ915188.

**References**

Thiers B. (continuously updated). Index Herbariorum: A global directory of public herbaria and associated staff. New York Botanical Garden's Virtual Herbarium. Available at: http://sweetgum.nybg.org/ih/.
