# Supplementary material for: Reconstructing the age and historical biogeography of the ancient flowering-plant family Hydatellaceae (Nymphaeales)
Source: BMC Evol Biol. 2014 May 13;14:102. doi: 10.1186/1471-2148-14-102 (PMC4030046; doi:10.1186/1471-2148-14-102)
Supplement: Additional file 3 — Chronogram and table of inferred ages from the Bayesian random local clock molecular dating analysis of the seed plants. [file 1471-2148-14-102-S3.doc]

**Additional file 3**. Chronogram and table of inferred ages from the Bayesian random local clock molecular dating analysis of the seed-plant data set.


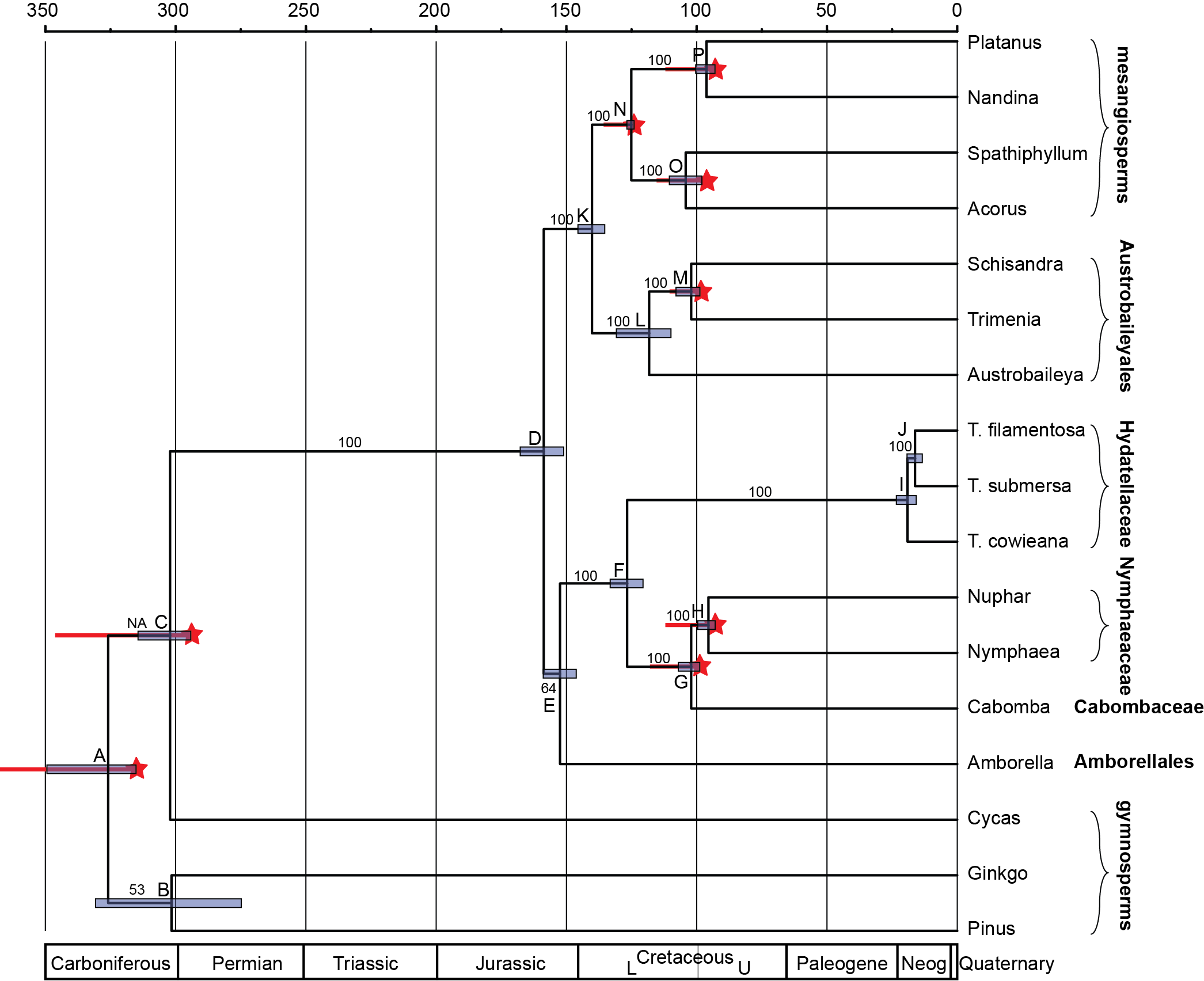


**Figure.** Bayesian random local clock dating of seed-plant phylogeny based on 13 plastid genes. Labelled nodes are cross-referenced with main text Table 1 and the Table below. The time scale is in Ma (geological time scale according the International Commission on Stratigraphy, 2012). Blue bars on nodes represent 95% HPD; red stars are minimum ages of calibration fossils (Table 1) and red bars are their assigned 95% prior age distributions. Numbers adjacent to branches are posterior probability support values (expressed as percentages). L, lower; Neog, Neogene; U, upper; T., *Trithuria*.

**Table.** Estimated ages of splits in seed-plant phylogeny based on a Bayesian random local clocks analysis of 13 plastid regions (see Figure above). Mean and 95% HPD are indicated for individual nodes labelled in the Figure above. All clades or taxa concern crown clades and taxa. MRCA, most recent common ancestor.

| Node | Clade or taxon | Mean age (Ma) | 95% HPD of age (Ma) |
| --- | --- | --- | --- |
| A | Seed plants | 325.87 | 315.21-349.37 |
| B | MRCA *Ginkgo* + *Pinus* | 301.56 | 274.79-330.71 |
| C | MRCA angiosperms + *Cycas* | 302.16 | 294.18-314.42 |
| D | Angiosperms | 158.67 | 151.05-167.72 |
| E | MRCA *Amborella* + Nymphaeales | 152.44 | 146.25-158.91 |
| F | Nymphaeales | 126.69 | 120.58-133.21 |
| G | MRCA *Cabomba* + Nymphaeaceae | 102.11 | 98.84-107.07 |
| H | Nymphaeaceae | 95.52 | 92.94-99.61 |
| I | Hydatellaceae | 19.07 | 15.72-23.36 |
| J | MRCA *T. filamentosa* + *T. submersa* | 16.21 | 13.41-19.28 |
| K | MRCA Austrobaileyales + Mesangiosperms | 140.22 | 135.32-145.58 |
| L | Austrobaileyales | 118.27 | 109.87-130.83 |
| M | MRCA *Schisandra* + *Trimenia* | 102.12 | 98.77-107.93 |
| N | Mesangiosperms | 125.11 | 124.06-126.82 |
| O | Monocots | 104.24 | 97.98-110.47 |
| P | Eudicots | 96.28 | 92.98-100.39 |
